# Frontal Cortex Transcriptome Analysis of Mice Exposed to Electronic Cigarettes During Early Life Stages

**DOI:** 10.3390/ijerph13040417

**Published:** 2016-04-12

**Authors:** Dana E. Lauterstein, Pamella B. Tijerina, Kevin Corbett, Betul Akgol Oksuz, Steven S. Shen, Terry Gordon, Catherine B. Klein, Judith T. Zelikoff

**Affiliations:** 1 Department of Environmental Medicine, New York University School of Medicine, Tuxedo, NY 10987, USA; del319@nyu.edu (D.E.L.); pbt217@nyu.edu (P.B.T.); kjc443@nyu.edu (K.C.); ShuQuan.Shen@nyumc.org (S.S.S.); terry.Gordon@nyumc.org (T.G); 2 Genome Technology Center, New York University School of Medicine, New York, NY 10016, USA; Betul.AkgolOksuz@nyumc.org; 3 Biochemistry and Molecular Pharmacology, New York University School of Medicine, New York, NY 10016, USA

**Keywords:** e-cigarettes, transcriptome, central nervous system, nicotine, development, RNA Sequencing, frontal cortex, pathway analysis

## Abstract

Electronic cigarettes (e-cigarettes), battery-powered devices containing nicotine, glycerin, propylene glycol, flavorings, and other substances, are increasing in popularity. They pose a potential threat to the developing brain, as nicotine is a known neurotoxicant. We hypothesized that exposure to e-cigarettes during early life stages induce changes in central nervous system (CNS) transcriptome associated with adverse neurobiological outcomes and long-term disease states. To test the hypothesis, pregnant C57BL/6 mice were exposed daily (via whole body inhalation) throughout gestation (3 h/day; 5 days/week) to aerosols produced from e-cigarettes either with nicotine (13–16 mg/mL) or without nicotine; following birth, pups and dams were exposed together to e-cigarette aerosols throughout lactation beginning at postnatal day (PND) 4–6 and using the same exposure conditions employed during gestational exposure. Following exposure, frontal cortex recovered from ~one-month-old male and female offspring were excised and analyzed for gene expression by RNA Sequencing (RNA-Seq). Comparisons between the treatment groups revealed that e-cigarette constituents other than nicotine might be partly responsible for the observed biological effects. Transcriptome alterations in both offspring sexes and treatment groups were all significantly associated with downstream adverse neurobiological outcomes. Results from this study demonstrate that e-cigarette exposure during early life alters CNS development potentially leading to chronic neuropathology.

## 1. Introduction

Information on e-cigarette usage patterns and their potential effects on adolescent and adult populations are rapidly emerging. However, few studies exist pertaining to usage and/or effects of e-cigarette use during pregnancy or their potential impacts on the unborn and young children. This issue needs more thorough investigation, since nicotine delivery during critical windows of brain development could lead to functional deficits, addiction, and/or serve as a gateway into further use of tobacco-containing products [1,2].

There is a general perception that e-cigarettes are safer than conventional cigarettes, and a growing number of pregnant women share this belief [3,4]. Approximately 10% of all women in the U.S. continue to smoke conventional cigarettes during pregnancy, and up to 40% of all women who smoke quit conventional cigarette use in preparation for and/or during pregnancy [5]. These “ex” smokers are particularly vulnerable to e-cigarette consumption, as they can perceive e-cigarette use as a “safer” way for pregnant women to maintain their ability to “smoke”. While not currently validated or confirmed scientifically, nicotine-replacement therapy products (e.g., nicotine patches and gum) appear to be less harmful to the fetus than conventional cigarettes or tobacco products and based on this, e-cigarettes could eventually prove to also be a “safer” alternative [2,6]. However, due to the lack of adequate safety assessments and toxicological data regarding the effects of every constituent in each new generation of e-cigarettes, safe use of these products during pregnancy remains uncertain. 

Although brain and central nervous system (CNS) development peaks during prenatal and neonatal time frames, the human brain continues to develop over the course of about two decades. Maternal use of conventional cigarettes during pregnancy has been positively associated with a number of adverse neurocognitive outcomes, including disruptive behavioral disorders and attention deficit hyperreactivity disorder [2,7]. Exposure during fetal development to nicotine, which passes the placental barrier and can bind to nicotinic acetylcholine receptors in the fetal nervous system that regulate brain maturation [8,9], can also interrupt and/or alter CNS maturation throughout the entire prenatal through young adult period creating the potential for a variety of adverse mental health outcomes [2]. 

Alterations in the human genome and epigenome have been linked with exposure to tobacco smoke [10]. Epigenome changes in early life can lead to alterations in gene expression that may increase disease risks later in life [11]. Such modifications have been related to the development of certain mental health disorders such as schizophrenia and major depressive disorder [12,13]. For example, modification of the gene for brain-derived neurotrophic factor (*BDNF*), an essential protein in the brain, which reinforces survival of existing neurons while simultaneously promoting neuronal growth and differentiation [14], has been associated with detrimental mental health outcomes [14]. Adolescents exposed *in utero* to maternal smoking show an increased DNA methylation of the *BDNF* gene promoter with previous studies indicating that a methylated *BDNF* gene acts as a diagnostic biomarker of depression [15,16]. While some studies have considered the toxicogenomic effects of cigarette smoke or nicotine exposure [17], the same information is currently not available for e-cigarettes.

Transcriptome profiling platforms allow for the thorough examination of gene expression patterns, and pathway analyses enables the prediction of biological outcomes associated with such changes. Ingenuity® Pathway Analysis (IPA) can provide calculated information on probable causal networks, as well as upstream and downstream effects from genome wide expression data by using previously determined cause-effect findings [18]. A major aim of this study was to utilize RNA-Seq to examine the transcriptome of frontal cortex brain samples from mice exposed via whole body inhalation to e-cigarette aerosols (with or without nicotine) during early life stages to identify those mental health issues that could be at higher risk later in life. The frontal cortex region was chosen because of its intergral role in executive function. 

## 2. Materials and Methods 

### 2.1. Animal Care and Exposures

Male and female C57BL/6 mice (Jackson Laboratory, Bay Harbor, ME, USA) were paired for 4 nights (2F/1M) at 9 weeks of age. After pairing, the males were removed, and females (2/cage) were exposed to e-cigarette aerosols (with or without nicotine) or filtered air for 3 h/day, 5 days/week via whole-body inhalation in 3 separate 1 m^3^ flow-through exposure chambers. Dams were separated into single cages and housed individually at or about gestational day (GD) 15, and daily exposures continued until parturition (~3 weeks). Subsequently, nursing dams and their pups were exposed together by whole-body inhalation starting on PND 4–6 and continuing throughout lactation (for a total of ~3 weeks). Offspring weights were measured each day preceding the exposures. Mice were housed in polycarbonate cages and different exposure polycarbonate cages were used for each exposure to minimize potential second- and third-hand exposures. During exposure, food and water were removed to avoid contamination with the aerosols; mice exposed to filtered air were handled identically to those exposed to e-cigarette aerosols. Cages were rotated in the chambers to ensure a uniform aerosol distribution to all animals throughout exposure. Chamber levels of total suspended particulates for the e-cigarette aerosols with or without nicotine, averaged 25.6 mg/m^3^ and 30.7 mg/m^3^, respectively. Concentration measurements were taken using a portable DataRAM 4™ (Thermo Scientific, Waltham, MA, USA). All animal procedures were conducted under New York University Institutional Animal Care and Use Committee (IACUC) approval. Animals were housed and treated in the NYU Animal Exposure Facility of the NYU NIEHS Center.

### 2.2. E-Cigarette Exposures

An automated 3-port e-cigarette aerosol generator (e~Aerosols, LLC, Central Valley, NY, USA) was used to produce e-cigarette aerosols from blu®, a popular and widely-used e-cigarette brand, classic tobacco flavor cartridges with or without nicotine. Each day a new nicotine (13–16 mg/mL) e-cigarette and a no-nicotine e-cigarette cartridge were used for exposures. Before use, the electrical resistance of each cartridge was measured to ensure the integrity of the heating coil, and the voltage was adjusted to produce a consistent wattage (~0.93 amperes) for each cartridge. The puff aerosols were generated with charcoal and HEPA filtered air using a rotorless and brushless diaphragm pump and the puff regime consisted of 35 mL puff volumes of 4-s duration at 30-s intervals. Each puff was mixed with filtered dilution air before entering the exposure chamber. Urinary cotinine levels in dams were measured using a commercially available ELISA kit (OraSure Technologies Inc., Bethlehem, PA, USA) to assure e-cigarette nicotine exposure in the appropriate treatment groups. Urine samples were obtained from 3 dams/treatment group at 2–3 h post-exposure on GD 16–19 and cotinine levels ranged between 664–1972 ng/mL; urinary cotinine levels were below detection limits for the no-nicotine e-cigarette and filtered air control groups. 

### 2.3. Sample Preparation

Four to six days after the last post-natal exposure, offspring at PND 25–31 were euthanized with pentobarbital (120 mg/kg) and brains were removed, sectioned, snap frozen in liquid nitrogen, and stored at −80 °C until RNA was extracted from the frontal cortex samples. A standard protocol for RNA isolation using TRIzol Reagent (Invitrogen) was used, and the RNA samples were subsequently purified using a RNeasy Mini Kit (Qiagen, Valencia, CA, USA). RNA was quantified using a NanoDrop ND-1000 fluorospectrometer (Thermo Scientific, Waltham, MA, USA). 

### 2.4. RNA-Seq, Ingenuity Pathway Analyses and Statistical Analyses

RNA libraries were prepared from the purified RNA samples for RNA-Sequencing using commercially available kits (Illumina, TruSeq® RNA Sample Prep Kit, San Diego, CA, USA). Samples (*n* = 6 offspring per treatment group/sex for a total of 36 samples; only one pup per litter was used for theses studies) were sequenced on the Illumina HiSeq2500 (San Diego, CA, USA) using HiSeq single read 50-cycle flow cells (Genome Technology Center (GTC), NYU Langone Medical Center). Male and female samples were sequenced with Illumina Hiseq 2500 platform in two separated runs and Illimuna bcl2fastq Conversion Software(version 1.8.4) was used to demultiplex the samples. Before processing data analysis, Fastqc (version 0.10.1, Basespace Labs Apps, Illumina, San Diego, CA, USA) was used to check the reads quality and PCR duplication. Reads were mapped to mm9 mouse transcriptome using Bowtie1 (version 0.12.9, maintained by John Hopkins University, Baltimore, MD, USA) with two mismatches allowed. The PCR duplicates were removed using Samtools (version 0.1.19, distributed under the Massachusetts Institute of Technology, Cambridge, MA, USA). Htseq (version 0.6.1.p.1,Python Software Foundation, Wilmington, DE, USA) was used to find the read counts for annotated genomic features. Principal Component Analysis was performed to detect variations among samples, in which one sample per sex group was detected as outlier and excluded from subsequent analysis. For the differential gene statistical analysis, DESeq2 R/Bioconductor package in the R statistical programming environment was used. Since male and female samples are prepared and sequenced in two different batches and batched effect is detected, we used one-way ANOVA to analyze the data separately instead of combining male and female data for two-way ANOVA analysis. In the analysis, the low counts were filtered with the Deseq2 algorithm if combined reads counts of retained samples for each gene was less than 5 per million. The Benjamini-Hochberg procedure (used R function *p* adjust) for multiple testing, which controls false discovery rate (FDR), was used to determine adjusted *p*-values. For each treatment group/sex dataset, which included (1) female offspring exposed to e-cigarettes with nicotine; (2) female offspring exposed to e-cigarettes without nicotine; (3) male offspring exposed to e-cigarettes with nicotine; and (4) male offspring exposed to e-cigarettes without nicotine, fold changes of genes with an adjusted *p*-value of <0.01 (when compared to air control values) were imported into IPA software to examine biological effects and disease pathway outcomes associated with the gene expression data. The RNA-Seq data have been deposited in the Gene Expression Omnibus, and are accessible through series accession number (GSE75858).

### 2.5. Real-Time Quantitative Polymerase Chain Reaction (qPCR)

Seven genes of interest selected from the RNA-Seq data were chosen for follow-up gene expression evaluation in male offspring frontal cortex samples using qPCR: nerve growth factor receptor (*Ngfr*), choline O-acetyltransferase (*Chat*), brain derived neurotrophic factor (*Bdnf*), glial cell line derived neurotrophic factor (*Gdnf*), galanin (*Gal*), T-box brain gene 1 (*Tbr1*), and alpha-1D adrenoreceptor (*Adra1d*). Commercially available TaqMan® gene expression assays for mice (Life Technologies, Grand Island, NY, USA) were used to prepare samples, which were processed using a QuantStudio 6K Flex Real Time PCR System (Applied Biosystems, Foster City, CA, USA). For each selected gene, the mRNA expression level was normalized to mRNA expression of the reference gene, glyceraldehyde-3-phosphate dehydrogenase (*Gapdh*).

### 2.6. Statistical Analysis for Obstetric Outcomes and qPCR

Biological parameters were analyzed by analysis of variance (ANOVA) followed by Bonferroni *post hoc* testing when appropriate. All statistical analyses were performed using GraphPad Prism® software (San Diego, CA, USA). 

## 3. Results

### 3.1. Effect of E-Cigarette Exposure on Neonatal Growth

**Figure 1:**
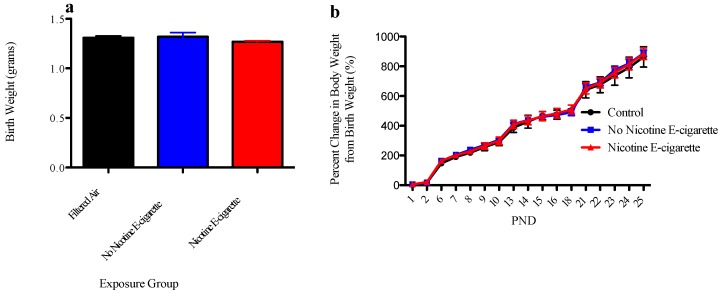
The effect of e-cigarettes on birth weight and weight gain. (**a**) Data represent average litter birth weights (*n* = 5–7 litters) ± SEM. Data were analyzed using a one-way ANOVA and Bonferroni *post-hoc* testing and data were not found to be significantly different from each other (*F*-value: 1.2, degrees of freedom: 2). (**b**) Data represent average litter weight gain (*n* = 3–5 litters) ± SEM. Data were analyzed using a two-way ANOVA and Bonferroni *post hoc* testing and were not found to be significantly different from each other (*F*-value: 0.91, degrees of freedom: 2).

Exposure to e-cigarette aerosols with and without nicotine did not alter birth weight or weight gain in offspring compared to their sex-matched control counterparts (Figure 1), suggesting that exposure (under these experimental conditions) did not result in overt malnourishment. 

### 3.2. Global Gene Expression Changes in Murine Frontal Cortex Samples from E-Cigarette Exposed Male and Female Offspring 

**Figure 2:**
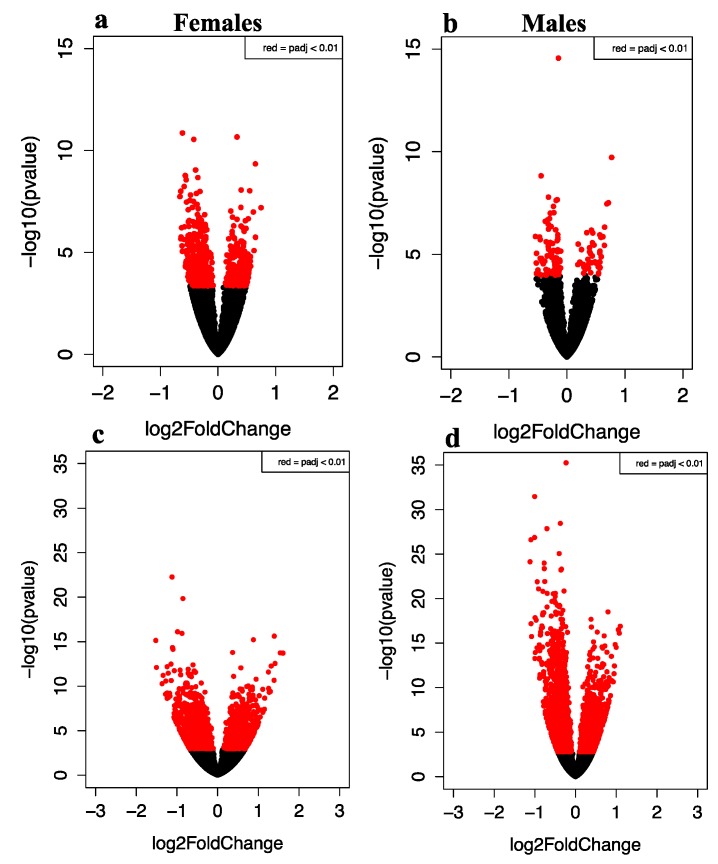
The effect of e-cigarettes with and without nicotine on global gene expression represented in terms of p-value and fold change (*n* = 5–6 frontal cortexes per each treatment and sex group). (**a**) Gene expression data for females exposed to e-cigarettes with nicotine (female controls = black, female e-cigarette with nicotine = red). (**b**) Gene expression data for males exposed to e-cigarettes with nicotine (male controls = black, male e-cigarette with nicotine = red). (**c**) Gene expression data for females exposed to e-cigarettes without nicotine (female controls = black, female e-cigarette without nicotine = red). (**d**) Gene expression data for males exposed to e-cigarettes without nicotine (male controls = black, male e-cigarette without nicotine = red).

**Figure 3:**
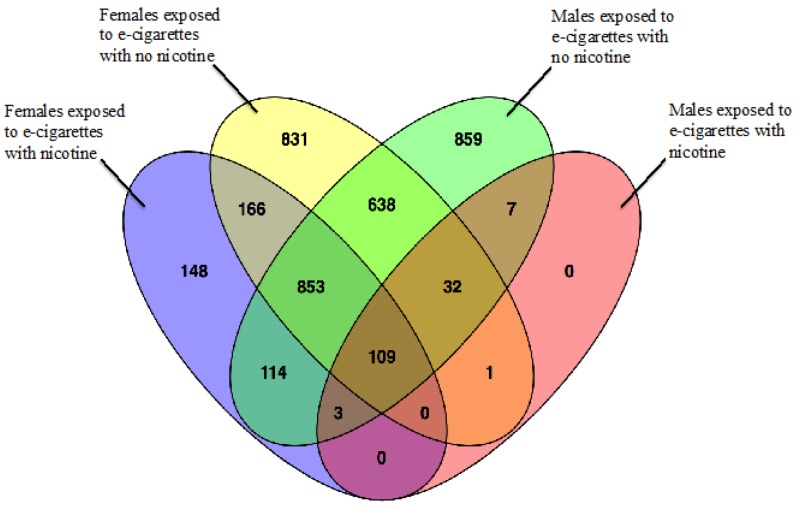
Quantity of overlapping and unique genes between treatment groups and sex. The Venn diagram displays overlapping and unique genes found to be significantly changed (*p* < 0.01) via RNA-Seq analysis between treatment groups and sex, as indicated. Diagram created using *Venny* [19]*.*

Early life repeated exposure to e-cigarette aerosols with or without nicotine induced alterations in the 1-month-old brain frontal cortex transcriptomes when compared to air-exposed control transcriptomes in both female and male offspring. As shown in the volcano plots for females (Figure 2a,c) and males (Figure 2b,d), genes that are highly dysregulated compared to controls are farther to the left and right sides of the plots, while highly significant gene expression changes appear higher on the plots. Frontal cortex brain samples from female offspring exposed pre- and post-natally to e-cigarette aerosols without nicotine had the greatest number of gene expression changes (2630) as seen in the yellow sector of the Venn diagram (Figure 3), whereas samples from the same brain region of the male offspring exposed to e-cigarette aerosols with nicotine (Figure 3, pink sector) demonstrated the least (152) gene expression changes compared to air exposed controls. 

For female offspring exposed to e-cigarette aerosol containing nicotine (Figure 3, blue sector), a total of 1393 genes were significantly changed (*p* < 0.01); whereas, only 152 genes were significantly changed (*p* < 0.01) compared to air exposed controls in males from the nicotine treatment group (Figure 3, pink sector). For female offspring exposed to e-cigarette aerosol with no nicotine, a total of 2630 genes were significantly changed (*p* < 0.01); a similar number of genes, 2615, were significantly changed (*p* < 0.01) in males from the same treatment group, of which 1632 were common between the two sexes. Comparisons among the different treatment groups revealed that offspring exposed to e-cigarette aerosol with no nicotine had the highest number of unique gene expression changes, while only 109 genes were commonly altered by all treatments in both sex groups (Figure 3, center brown sector). These data suggest common factors between treatment and sex that could be driving common gene expression alterations. 

### 3.3. IPA Predicted Downstream Disease and Biological Function Effects Associated with the Observed Gene Expression Changes

Using IPA, a core analysis was completed to examine the top five predicted disease and disorder categories for each treatment group and sex (Table 1). Female offspring exposed to e-cigarette aerosols with or without nicotine, and male offspring exposed to e-cigarette aerosols without nicotine had the same predicted disease and disorder outcomes that included cancer, organismal injury and abnormalities, neurological disease, psychiatric disorders, and gastrointestinal disease. However, the number of molecules demonstrating altered gene expression involved, and the p-values differed for each group. The male offspring exposed to e-cigarettes with nicotine had three of five outcomes in common (*i.e.*, cancer, organismal injury and abnormalities, and gastrointestinal disease), but also included dermatological diseases and conditions, and connective tissue disorders. 

**Figure 4:**
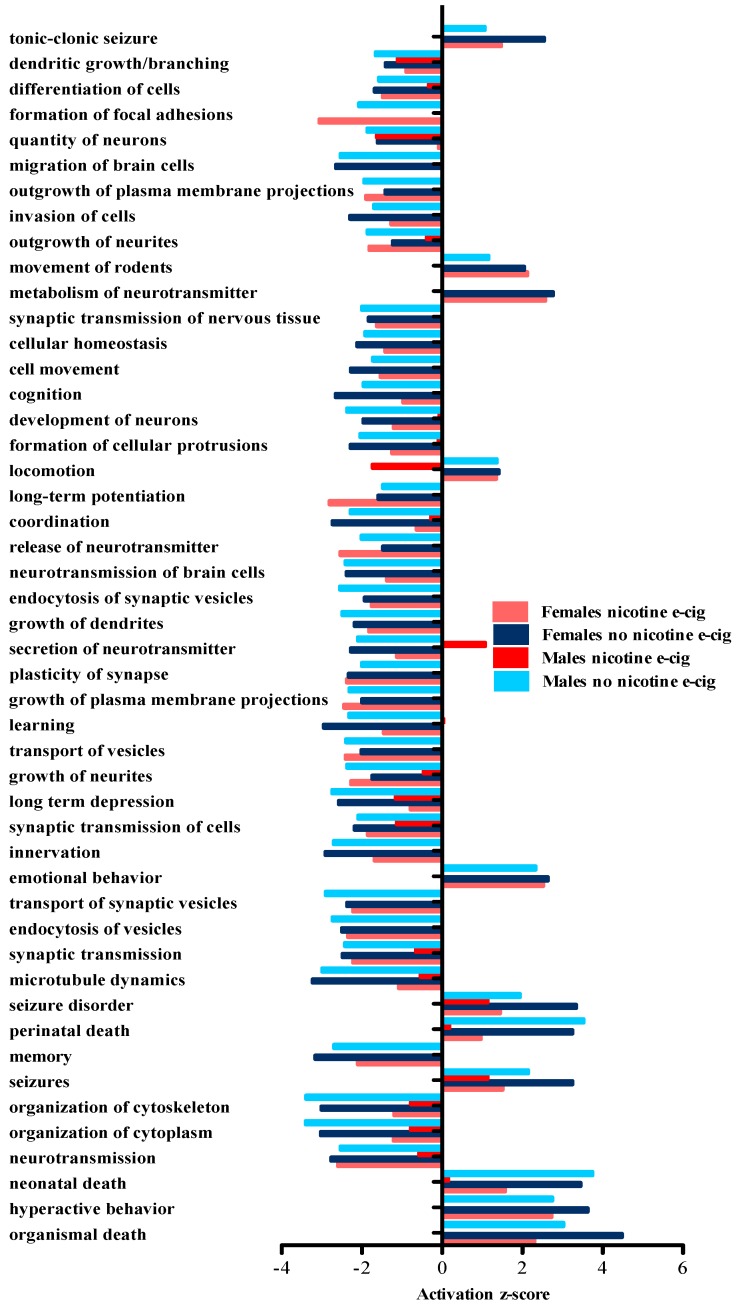
Top 50 disease and biological functions predicted to be altered using IPA comparison analysis. Data obtained from RNA-Seq were filtered to only include those results reaching a *p* < 0.01 significance level; these RNA-Seq data were then imported into IPA for comparison analysis. A positive activation z-score reflects induction of the indicated disease/biological function, while a negative z-score reflects inhibition of the indicated disease/biological function. Z-scores of +/− 2.0 were considered significant.

IPA was also used to carry out a comparison analysis between all the e-cigarette exposed groups. This type of analysis allowed for identification of disease or biological functions that are predicted to have similar changes across treatment group and sex. The IPA-generated heat map data were exported, and the top 50 disease and biological functions were examined further (Figure 4). Included in the predominant predicted disease and biological functions revealed were: decreases in memory, cognition, learning and neurotransmission, and increases in hyperactive behavior, emotional behavior and organismal death (seen in all groups except male offspring exposed early in life to e-cigarettes containing nicotine). IPA analyses also revealed predicted decreases in dendritic growth/branching and quantity of neurons, as well as predicted increases in seizure disorder and seizures in all treatment groups and both sexes exposed to e-cigarettes with and without nicotine. In general, all treatment groups tended to show the same directionality (inhibition or activation) for reported outcomes. In contrast, male offspring exposed to e-cigarettes containing nicotine demonstrated a predicted decrease in locomotion, while the other groups showed a predicted increase. In addition, the same male treatment group revealed a predicted increase in secretion of neurotransmitters in contrast to the other exposure groups, which showed a predicted decrease.

### 3.4. Follow-Up of Gene Expression Analysis of Selected Genes by qPCR in Male Frontal Cortex Samples

Following RNA-Seq analysis, seven genes of interest, *Ngfr*, *Chat*, *Bdnf*, *Gdnf*, *Gal*, *Tbr1*, and *Adra1d* were selected for follow-up analysis with qPCR to compare to the RNA-Seq data. These particular genes were selected because of their relevance to neurological disorders/mental health. In general, gene expression data obtained from qPCR analysis showed similar trends (e.g., directionality and fold change) to those revealed by RNA-Seq (Table 2). Any dissimilarities observed between the two analyses were likely due to differences in platform sensitivity.

## 4. Discussion

The present study investigated effects of early life exposure to e-cigarette aerosols, with and without nicotine on CNS development by examining gene expression in the developing brains of exposed mice. This investigation sought to determine whether, and to what extent, early life exposure to aerosols produced by e-cigarettes affected neurodevelopment leading to gene expression changes that could result ultimately in cognitive or behavioral deficits later in life. Utilizing next-generation sequencing, gene and gene pathway analyses were performed on brain frontal cortex samples to assess any e-cigarette-induced changes in gene expression networks. This approach allowed for the examination of global gene expression changes, as well as facilitated the prediction of neurobiological downstream effects by IPA. 

Toxicological data concerning e-cigarettes are extremely limited and even less so regarding effects on the developing fetus and neonate. Though few data exist concerning the effects of early life exposure to e-cigarette aerosols on neurodevelopment, a plethora of information is available concerning the effects of early life exposure to cigarette smoke and nicotine alone. It is well documented that fetal, childhood, and adolescent exposure to cigarette smoke is associated with adverse neurocognitive and neurobehavioral outcomes including decreases in general intellectual ability and auditory learning, development of conduct disorders, and increased hyperactivity-impulsivity behaviors [20,21,22]. Nicotine is believed to be the principal constituent of cigarette smoke that drives adverse neurodevelopmental outcomes from gestational exposure [20,23,24]. However, while prenatal exposure to nicotine is known from animal studies to produce adverse neurobehavioral consequences, underlying mechanisms for such a finding have not yet been established [2]. Thus, as nicotine is a primary constituent of e-cigarettes, it is conceivable that exposure to the aerosols during early life could induce neurodevelopmental alterations similar to those produced by exposure to conventional cigarettes. In fact, a recent study in mice reported that exposure late *in utero* and during early postnatal life to aerosols from e-cigarettes containing nicotine lead to later life behavioral changes (*i.e.*, increased locomotor activity and increased cognitive flexibility) in adult male mice [25]. As increases in mental and behavioral disorders are a universal public health concern, there is an urgent need for continued investigations into emerging nicotine-associated products such as e-cigarettes. 

In this study, gene expression changes were stratified by treatment and offspring sex in order to evaluate downstream diseases, disorders, and biological functions by IPA. Individual core and comparison analysis of the exposure groups yielded similar results, suggesting that although individual gene changes differed between treatment groups, common gene networks were also altered by e-cigarettes (regardless of nicotine content or sex) leading to comparable downstream effects. Female offspring exposed to e-cigarettes with or without nicotine, and male offspring exposed to e-cigarettes without nicotine all revealed neurological disease and psychiatric disorders in the top five IPA predicted disease/disorders retrieved through core analysis. When examined by comparison analysis, these same groups showed similar changes including decreases in memory, cognition, and learning.

Similar gene expression changes were observed here in female offspring exposed to e-cigarettes (with and without nicotine) as those seen in male offspring exposed to e-cigarettes without nicotine. In contrast, male offspring exposed to e-cigarettes with nicotine exhibited a smaller number of gene expression changes, as well as different IPA-predicted diseases and disorders. A possible explanation for these differences observed in the male nicotine-exposed offspring is the inhibitory action of nicotine on aromatase, a required enzyme for estrogen biosynthesis that converts androgens to estrogens; lack of this enzyme results in estrogen deficiency [26]. Estrogen deficiency is known to have adverse effects in males including insufficiencies in skeletal maturation and sexual behavior [27,28]. It is likely that an estrogen deficiency, and thus probable masculinization of the brain, would thus have a greater effect in females (compared to controls). In this study, mice offspring were sacrificed at the initiation of puberty, *i.e.*, between PND 25–31. This period is marked by dramatic changes in gonadal hormones [29], which in combination with nicotine-induced aromatase inhibition could result in major differences in gene expression between females and males exposed to nicotine. This notion is supported by the studies of Cao et al (2013) who examined the effects of gestational nicotine exposure on genes involved in brain myelination of adolescent rats sacrificed at PND 35 [29]. Findings from the aforementioned study also revealed sex-dependent differences in the prefrontal cortex of male and female rats, such that gestational nicotine exposure enhanced myelination processes in males (*i.e.*, positive fold-changes in gene expression), but decreased the same processes in females (negative fold-changes in gene expression) [29]. In aromatase knock-out (ArKO) adult mice, wild-type females demonstrated depressive behaviors, while the ArKO−/− males did not [26,30,31]. Other studies using rodents have also documented that prenatal nicotine exposure increases depression and anxiety behaviors in female offspring, but not in male offspring [26,32,33,34]. Further investigation is required to delineate the mechanisms behind these differences.

Based on the neurotoxic effects of nicotine during neurodevelopment, we hypothesized that exposure to e-cigarettes with nicotine would produce the greatest number of transcriptomic changes in the offspring. Surprisingly, studies here demonstrated that early life exposure to e-cigarette aerosols without nicotine produced the greatest number of significant gene expression changes in frontal cortex samples and IPA-predicted alterations in biological functions/disease outcomes in offspring of both sexes. These results suggest that e-cigarette components other than nicotine could also play an important role in affecting neurodevelopment. However, due to the lack of inhalation exposure data, it is difficult to identify the role of each individual e-cigarette liquid component, other than nicotine, when interpreting the results. 

E-cigarette liquid is typically a mixture of propylene glycol, glycerol, nicotine, flavorings, and other additives [35,36]. The blu® classic tobacco flavored disposable e-cigarette liquid used for the studies here has been shown to produce an aerosol containing 73% glycerin and/or propylene glycol, 15% water, 11% flavorings, and 1% nicotine (package reported 24 mg nicotine/unit) [37]. Propylene glycol and glycerol, two of the main ingredients in e-cigarette liquids, are “generally regarded as safe” (GRAS) when ingested, but information concerning effects of these agents via an inhalation exposure route is severely lacking [36,38]. In studies where propylene glycol was used to create a fog mist in the theatre industry, resulting in both inhaled and dermal contact, extensive airway and eye irritation, respectively were reported [39]. Prolonged or repeated inhalation of propylene glycol has also been reported to affect the CNS and behavior [39]. Detailed information regarding the flavorings is not available, although it is likely that an aerosol produced from classic tobacco cartridges without nicotine would have a similar chemical composition, other than nicotine content, to the aerosol produced from a cartridge with nicotine.

Based on our results demonstrating that early life exposure to non-nicotine containing aerosols produce greater frontal cortex gene expression changes, it is likely that one or more of the aforementioned e-cigarette constituents, besides nicotine, could be driving the observed effects. These findings emphasize the need for further studies on e-cigarette liquid constituents using a relevant exposure route. This need is stressed by the recent finding that diacetyl, a flavoring substance that is also considered GRAS by ingestion, and acetyl propionyl, a structurally similar flavoring to diacetyl, have both been found in a number of e-cigarette solutions, including those claimed by the manufacturer to not be present [36,40], particularly since diacetyl has been implicated as a causative agent in the development of bronchiolitis obliterans, an irreversible lung disease commonly referred to as “popcorn lung” [36,40,41,42]. Additionally e-cigarettes have been shown to emit metals such as nickel, zinc and silver [43]. Overall, there appears to be a critical need for improved quality control of these products and toxicological assessment of individual e-cigarette liquid constituents via inhalation.

## 5. Conclusions 

E-cigarettes continue to invoke controversy among the general public and in scientific communities around the world. This study is the first (to our knowledge) to examine global gene expression changes in the developing brain induced by e-cigarette aerosols. Results from this study demonstrate that e-cigarette aerosols, both with and without nicotine, induce sex-dependent gene expression changes associated with predicted adverse neurobiological and neurobehavioral outcomes similar to those associated with early life exposure to the smoke from conventional cigarettes. Behavioral testing is needed to correlate the adverse neurobiological and neurobehavioral predicted outcomes with functional outcomes, and lack of these studies are a limitation of this study. However, future behavioral studies are planned to test these predictions.

Recent and emerging studies that compare the toxicities of e-cigarettes to conventional cigarettes demonstrate that e-cigarettes may indeed be the less harmful choice [37,44], most likely due to the elimination of overt combustion products. While the use of e-cigarettes may prove to be a “reduced harm” or “cessation aid” product for conventional adult cigarette/tobacco users, they could pose a substantial threat to the developing CNS if exposure occurs during prenatal, childhood and adolescent life stages. Thus, e-cigarettes are a public health risk that needs to be further examined and addressed through appropriate research, regulation and policy considerations. 

## Figures and Tables

**Table 1:** Top 5 Ingenuity® Pathway Analysis (IPA) disease and disorder categories for each treatment group and sex compared to sex-matched air controls.

**Female Offspring Exposed to E-Cigarette Containing Nicotine**		
Ingenuity Disease and Disorders	*p*-Value	# of Molecules
Cancer	5.43 E−05–2.09 E−28	1157
Organismal Injury and Abnormalities	7.31 E−05–2.09 E−28	1162
Neurological Disease	7.75 E−05–1.78 E−27	484
Psychiatric Disorders	7.00 E−05–1.33 E−22	320
Gastrointestinal Disease	5.85 E−05–1.26 E−21	838
**Female Offspring Exposed to E-Cigarette without Nicotine**		
Ingenuity Disease and Disorders	*p*-Value	# of Molecules
Cancer	6.03 E−06–3.98 E−46	2121
Organismal Injury and Abnormalities	6.82 E−06–3.98 E−46	2130
Neurological Disease	8.19 E−06–1.55 E−35	811
Gastrointestinal Disease	6.03 E−06–3.33 E−35	1553
Psychiatric Disorders	8.19 E−06–5.77 E−31	537
**Male Offspring Exposed to E-Cigarette Containing Nicotine**		
Ingenuity Disease and Disorders	*p*-Value	# of Molecules
Cancer	1.38 E−02–2.18 E−12	137
Organismal Injury and Abnormalities	1.38 E−02–2.18 E−12	137
Dermatological Diseases and Conditions	6.92 E−03–9.47 E−11	80
Connective Tissue Disorders	6.92 E−03–1.73 E−10	83
Gastrointestinal Disease	1.38 E−02–7.37 E−06	99
**Male Offspring Exposed to E-Cigarette without Nicotine**		
Ingenuity Disease and Disorders	*p*-Value	# of Molecules
Cancer	2.97 E−06–2.20 E−35	2096
Organismal Injury and Abnormalities	2.97 E−06–2.20 E−35	2108
Neurological Disease	3.27 E−06–7.20 E−34	814
Gastrointestinal Disease	1.10 E−06–1.27 E−30	1516
Psychiatric Disorders	3.27 E−06–1.34 E−27	510

The number of molecules, calculated from the gene expression data, indicates how many molecules/genes in the disease/disorder pathway were altered. The *p*-value range indicates the range of significance for the molecules involved in the pathway.

**Table 2:** Comparison of gene expression data from qPCR and RNA-Seq analyses. Gene expression fold-change and *p*-values for frontal cortex samples recovered from male offspring exposed early in life to e-cigarette aerosols with or without nicotine are compared using qPCR and RNA-Seq.

Gene	Frontal Cortex Samples from Male Offspring Exposed to E-Cigarettes Containing Nicotine				Frontal Cortex Samples from Male Offspring Exposed to E-Cigarettes Without Nicotine			
	RNA-Seq		qPCR		RNA-Seq		qPCR	
	Fold Change	Adjusted *p*-Value	Fold Change	*p*-Value	Fold Change	Adjusted *p*-Value	Fold Change	*p*-Value
Ngfr	2.37	3.94E−3	2.82	NS	3.16	1.57E−6	4.36	0.05
Chat	1.88	6.38E−4	2.17	NS	2.66	9.71E−11	3.31	NS
Bdnf	−1.22	NS	−0.91	NS	−1.67	1.81E−5	−1.55	NS
Gdnf	ND	NS	1.47	NS	1.93	3.6E−5	2.13	NS
Gal	ND	NS	5.6	0.001	3.66	2.01E−6	9.56	0.001
Tbr1	−1.31	NS	−1.25	NS	−2.02	1.68E−11	−1.91	NS
Adra1d	−1.47	NS	−2.21	NS	−2.29	9.86E−5	−3.05	0.05

Data from qPCR ( *n* = 3–6 frontal cortex samples/treatment group; only one pup per litter was used for these studies) was analyzed using a two-way ANOVA and Bonferroni *post hoc* testing (for treatment factor, *F*-value: 2.08, degrees of freedom: 2; for gene factor, *F*-value: 26.6, degrees of freedom: 6). ND = Not Detected, NS = Not Significant.

## Abbreviations

The following abbreviations are used in this manuscript:

E-cigarettes: Electronic cigarettes
CNS: central nervous system
PND: postnatal day
RNA-Seq: RNA Sequencing
IPA: Ingenuity® Pathway Analysis
GD: gestational day
GRAS: generally regarded as safe
Ngfr: nerve growth factor receptor
Chat: choline O-acetyltransferase
Bdnf: brain derived neurotrophic factor
Gdnf: glial cell line derived neurotrophic factor
Gal: galanin
Tbr1: T-box brain gene 1
Adra1d: alpha-1D adrenoreceptor
Gapdh: glyceraldehyde-3-phosphate dehydrogenase
